# Docetaxel-Induced Cell Death Is Regulated by a Fatty Acid-Binding Protein 12-Slug-Survivin Pathway in Prostate Cancer Cells

**DOI:** 10.3390/ijms25179669

**Published:** 2024-09-06

**Authors:** Rong-Zong Liu, Mansi Garg, Xiao-Hong Yang, Roseline Godbout

**Affiliations:** Department of Oncology, Cross Cancer Institute, University of Alberta, Edmonton, AB T6G 1Z2, Canada; rongzong@ualberta.ca (R.-Z.L.); xyang@ualberta.ca (X.-H.Y.)

**Keywords:** fatty acid-binding protein 12, chemoresistance, docetaxel, apoptosis, Survivin

## Abstract

Chemotherapy is an important treatment option for advanced prostate cancer, especially for metastatic prostate cancer (PCa). Resistance to first-line chemotherapeutic drugs such as docetaxel often accompanies prostate cancer progression. Attempts to overcome resistance to docetaxel by combining docetaxel with other biological agents have been mostly unsuccessful. A better understanding of the mechanisms underlying docetaxel resistance may provide new avenues for the treatment of advanced PCa. We have previously found that the fatty acid-binding protein 12 (FABP12)-PPARγ pathway modulates lipid-related bioenergetics and PCa metastatic transformation through induction of Slug, a master driver of epithelial-to-mesenchymal transition (EMT). Here, we report that the FABP12-Slug axis also underlies chemoresistance in PCa cells. Cell sensitivity to docetaxel is markedly suppressed in FABP12-expressing cells, along with induction of Survivin, a typical apoptosis inhibitor, and inhibition of cleaved PARP, a hallmark of programmed cell death. Importantly, Slug depletion down-regulates Survivin and restores cell sensitivity to docetaxel in FABP12-expressing cells. Finally, we also show that high levels of Survivin are associated with poor prognosis in PCa patients, with FABP12 status determining its prognostic significance. Our research identifies a FABP12-Slug-Survivin pathway driving docetaxel resistance in PCa cells, suggesting that targeting FABP12 may be a precision approach to improve chemodrug efficacy and curb metastatic progression in PCa.

## 1. Introduction

Prostate cancer (PCa) is the most prevalent cancer in men. While localized PCa can usually be cured, treatment options are limited for metastatic disease [1]. Androgen blocking is the basis of initial therapy for metastatic PCa. Progression of metastatic castration-resistant PCa, however, is almost inevitable. As such, docetaxel, a member of the taxane family and a potent chemotherapeutic drug, has been used as first-line standard of care for metastatic castration-resistant PCa patients. Unfortunately, the development of resistance to docetaxel is common, and the underlying mechanisms appear to be diverse and remain to be understood.

Docetaxel shares a common mechanism of action with paclitaxel but shows much more potent cytotoxicity compared to paclitaxel [2]. It promotes and stabilizes microtubule assembly, thus disrupting microtubule dynamics, which leads to inhibition of mitotic cell division and initiation of cell apoptosis [3,4]. Despite being an important first-line chemotherapy drug conferring excellent initial response in many cancers including PCa, docetaxel is prone to cellular drug resistance via a variety of different mechanisms [5,6]. For instance, androgen signaling, drug efflux and influx mediators, and stem cell features have all been found to contribute to increased resistance to docetaxel in PCa [5]. In addition, there is accumulating evidence showing that aberrant lipid metabolism [7] and epithelial-to-mesenchymal transition (EMT)-mediated metastasis [8,9] play important roles in docetaxel resistance. It has long been known that PCa depends on lipids, rather than glucose commonly used by other cancers, to sustain energy demands associated with tumor growth and dissemination [10,11]. Aberrant lipid metabolism has been deemed a hallmark of PCa progression and treatment resistance [12,13].

FABP12, the most recently-identified member of the fatty acid-binding protein family [14], is preferentially amplified and upregulated in metastatic PCa, and promotes lipid droplet accumulation, mitochondrial β-oxidation and EMT in PCa cells [15]. FABP12 functions through the transcription factor ‘peroxisome proliferator-activated receptor gamma’ (PPARγ) [15], a fatty acid-activated nuclear receptor and driver of metastasis in PCa [16,17]. Here, we examine a possible role for FABP12 in PCa cell resistance to docetaxel. We identify the apoptosis inhibitor Survivin as a downstream effector of FABP12 and demonstrate a functional link between the FABP12-Slug-Survivin axis and resistance of PCa cells to docetaxel through reduced apoptosis.

## 2. Results

### 2.1. Fatty Acid-Binding Protein 12 (FABP12) Suppresses Docetaxel-Induced Cell Growth Inhibition

**Figure 1:**
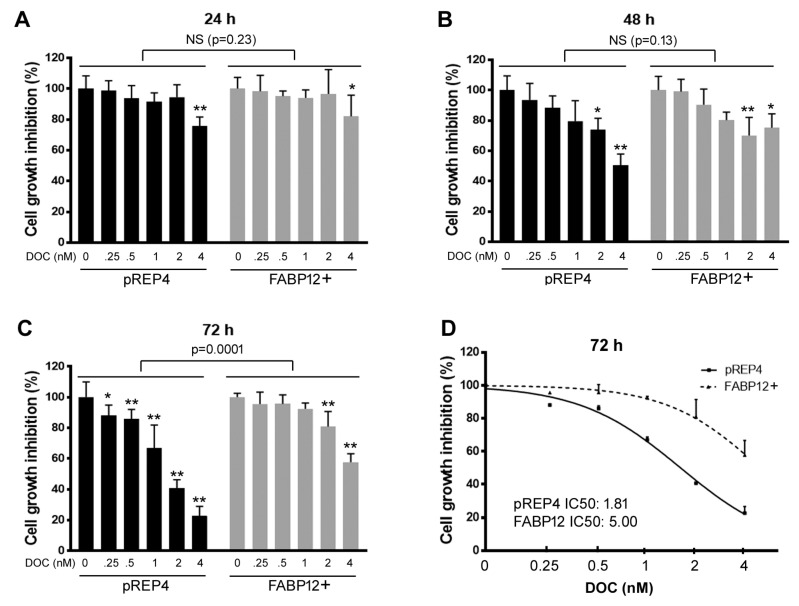
Cell growth suppression by docetaxel in PC3 stable transfectants. PC3 cells stably transfected with empty vector (pREP4) or pREP4 containing FABP12 cDNA encoding the entire FABP12 open reading frame (FABP12+) were cultured in 96-well plates in the absence (control) or presence of docetaxel (DOC, concentrations as indicated) for 24 (**A**), 48 (**B**) or 72 (**C**) hours. Cells were then stained with crystal violet and cell density analyzed with a microplate reader. Six wells were used for each concentration of docetaxel with two of the six wells left unstained and used as background controls. (**D**) IC_50_ for PC3-pREP4 and FABP12+ cells based on cell growth curves as a function of docetaxel treatment. Curves were generated with MS Excel. Each experiment was repeated at least three times. *, *p* < 0.05; **, *p* < 0.01.

We have previously shown that the FABP12-PPARγ pathway in PCa cells triggers epithelial-to-mesenchymal transition (EMT), a critical process involved in tumor progression, metastatic transformation and treatment resistance. To further explore the role of FABP12 in PCa treatment, we examined the effect of FABP12 expression on cell growth inhibition in PC3 cells treated with docetaxel. As FABP12 is naturally expressed at low levels in PCa cell lines, we generated clonal populations of PC3 cells with ectopic expression of FABP12 using the pREP4 episomal vector [15]. PC3 cells transfected with either empty vector (pREP4) or the pREP4-FABP12 expression construct (FABP12+) were treated with increasing concentrations (0, 0.25, 0.5, 1, 2, 4 nM) of docetaxel. After 24 h of treatment, control and FABP12+ cells only showed a response to the highest concentration of docetaxel (4 nM), with no difference in cell growth noted between control and FABP12+ cells (*p* = 0.23) (Figure 1A). After 48 h, both the pREP4 and FABP12+ cell lines showed significant growth inhibition at 2 and 4 nM docetaxel. Although no overall difference was noted between the two cell lines (*p* = 0.13), 4 nM docetaxel had a stronger effect on control pREP4 cells compared to FABP12+ cells (Figure 1B). At 72 h, significant cell growth inhibition was observed at all doses of docetaxel tested in control cells; however, FABP12+ cells only showed significant responses at the higher concentrations of docetaxel (2 and 4 nM) (Figure 1C). Overall, FABP12 expression had a significant effect (*p* = 0.0001) on docetaxel-induced cell growth inhibition, with IC50s of 1.81 nM and 5.00 nM for pREP4 control and FABP12+ cells, respectively (Figure 1D). The effects of FABP12 expression on docetaxel-induced cell growth inhibition were not significant at the earlier time points. These results indicate a suppressive, albeit delayed, role for FABP12 in PC3 cell sensitivity to docetaxel. 

### 2.2. The Slug-Survivin Pathway Underlies the Role of Fatty Acid-Binding Protein 12 (FABP12) in Cell Growth 

**Figure 2:**
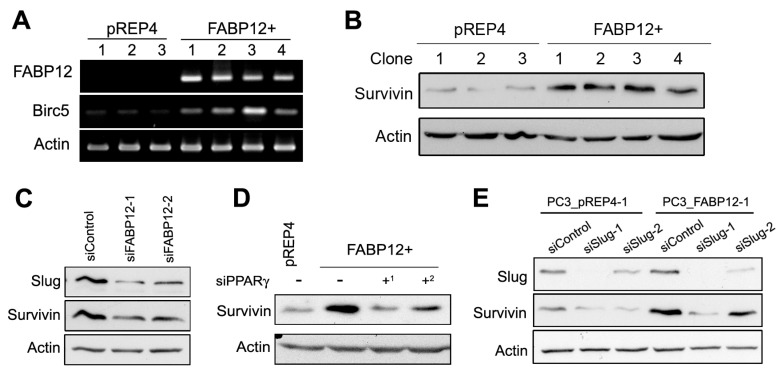
Western blot analysis of the FABP12-Slug-Survivin pathway in mediating PC3 cell sensitivity to docetaxel. (**A**,**B**) Semi-quantitative RT-PCR analysis of *BIRC5* RNA (**A**) and Western blot analysis of Survivin protein (**B**) in stable transfectants PC3-pREP4 (pREP4) (three independent clones) and PC3 pREP4-FABP12 (FABP12+) (four independent clones). (**C**) Western blot showing reduction in levels of Survivin upon FABP12 knockdown in PC3-FABP12+ cells using two different FABP12 siRNAs (siFABP12-1 and siFABP12-2). (**D**) Western blot showing reduced levels of Survivin in PC3-FABP12+ cells transfected with two different PPARγ siRNAs: siPPARγ-1 (^1^) and siPPARγ-2 (^2^). (**E**) Western blot showing reversal of FABP12-mediated induction of Survivin upon depletion of Slug using two different siRNAs (siSlug-1 and siSlug-2). Actin was used as the loading control.

The growth of cancer cells is regulated by molecular mechanisms governing cell cycle progression, cell survival, and cell death, all of which are intimately related and are known to be affected by chemotherapeutic drug treatment. Survivin, encoded by the gene *BIRC5* [Baculoviral IAP (inhibitor of apoptosis) Repeat Containing 5], is a prominent inhibitor of apoptosis and regulator of cell proliferation during normal development as well as in human cancers [18,19]. As Survivin is known to regulate chemodrug sensitivity in cancer cells [20,21], we investigated whether Survivin could be a downstream effector of FABP12 in modulating cellular response to docetaxel. We first examined the levels of *BIRC5* mRNA in three independent PC3 control and four independent PC3-FABP12 stable transfectants. We observed induction of *BIRC5* expression in all four PC3-FABP12+ cell lines compared to the PC3 control cell lines (Figure 2A). Western blot analysis of the same PC3 cell lines confirmed the induction of Survivin in all four PC3-FABP12+ cell lines compared to PC3 control cell lines (Figure 2B). Depletion of FABP12 in PC3-FABP12+ cells resulted in marked reduction in Slug and Survivin levels (Figure 2C), extending the previously reported regulatory relationship between FABP12 and Slug, to Survivin. 

We previously found that the EMT-promoting transcription factor Slug is induced upon ectopic expression of FABP12 in PCa cells [15]. As FABP12 activates PPARγ, a fatty acid-activated nuclear transcription factor, and PPARγ knockdown significantly reduces Slug levels [15]; we next examined a possible relationship between PPARγ and Survivin by knocking down PPARγ in FABP12+ cells. PPARγ knockdown resulted in considerably reduced Survivin levels (Figure 2D). As Slug knockdown also reduced Survivin levels, especially in FABP12+ cells (Figure 2E), our data suggest a FABP12-PPARγ-Slug-Survivin regulatory axis in PCa cells. 

### 2.3. Depletion of Slug Restores Docetaxel Sensitivity in Fatty Acid-Binding Protein 12+ (FABP12+) Cells 

**Figure 3:**
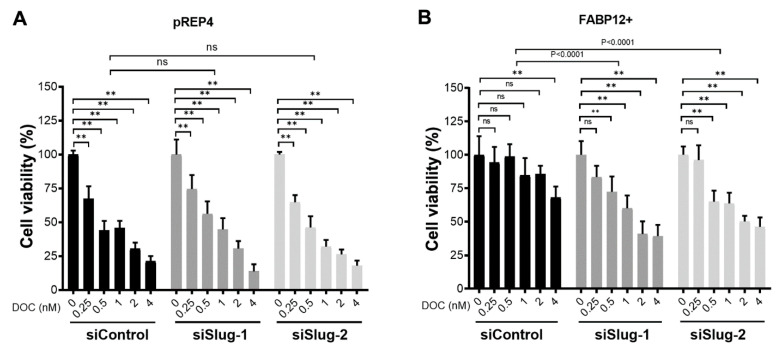
Slug depletion increases the sensitivity of PC3-FABP12+ cells to docetaxel. PC3-pREP4 (pREP4) cells (**A**) and PC3-FABP12 (FABP12+) cells (**B**) were transfected with scrambled siRNAs (siControl) or siRNAs targeting Slug (siSlug-1, siSlug-2) and treated with docetaxel at the indicated concentrations for 72 h. Cell growth was analyzed by crystal violet staining; ns denotes not significant; **, *p* < 0.01. Experiments were repeated three times.

We have previously shown that FABP12 induces EMT in PCa cells through induction of Slug, a master transcription factor for EMT transformation [22]. We therefore asked whether Slug might also serve as a downstream effector of FABP12 in PCa cell response to docetaxel. To this end, we performed siRNA knockdown of Slug in PC3 control and FABP12+ cell lines, and then treated these cells with increasing concentrations of docetaxel. As expected, FABP12-mediated suppression of docetaxel-induced cell growth inhibition was observed in PC3 cells transfected with control siRNAs (panels with black bars in Figure 3A,B). Depletion of Slug by two different siRNAs had no effect on docetaxel sensitivity in pREP4 cells (panels with gray bars in Figure 3A); however, docetaxel-induced cell growth inhibition was significantly restored after Slug depletion in FABP12+ cells (siSlug-1, *p* < 0.0001; siSlug-2, *p* < 0.0001) (panels with gray bars in Figure 3B). These results are in keeping with Slug mediating PCa cell response to docetaxel through FABP12.

### 2.4. Docetaxel Induces Fatty Acid-Binding Protein 12 (FABP12)-Dependent Prostate Cancer (PCa) Cell Apoptosis 

**Figure 4:**
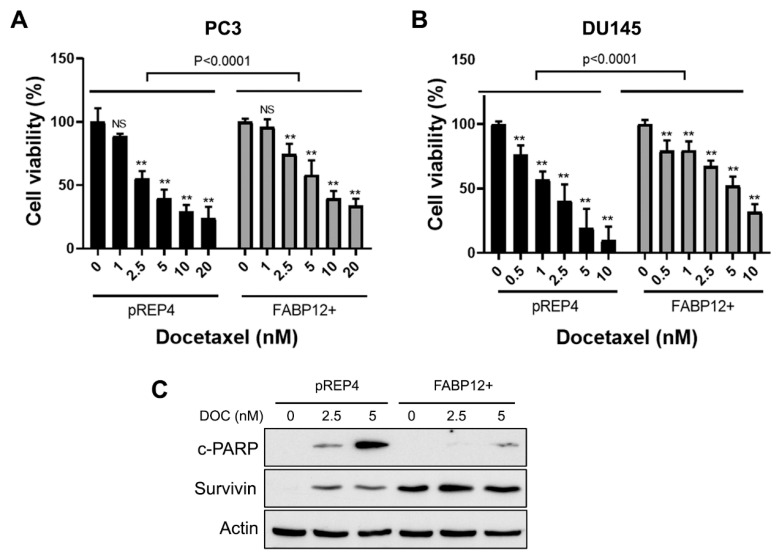
Analysis of the modulation of docetaxel-induced cell death by FABP12 in PCa cells. (**A**,**B**) Control (pREP4) and FABP12+ PC3 (**A**) and DU145 (**B**) cells were treated with docetaxel (DOC) at the indicated concentrations for 72 h. Cell viability was analyzed with the MTS assay kit (Promega). (**C**) Western blots showing the effects of FABP12 expression and docetaxel treatment on the expression of the apoptosis marker cleaved-PARP (c-PARP) and Survivin in DU145 stable transfectants. All docetaxel treatment experiments were repeated at least three times; ns denotes not significant; **, *p* < 0.001.

Survivin is an inhibitor of apoptosis and a major factor in determining docetaxel efficacy in cancer cells [20,21]. In light of the functional link between FABP12 and Survivin expression, we next asked whether FABP12 might protect PCa cells from docetaxel-induced apoptotic cell death. We first examined cell viability as a function of docetaxel treatment in pREP4 and FABP12+ PC3 cells using the MTS assay as a surrogate for cell viability. Cells were treated with increasing concentrations of docetaxel for 72 h. While docetaxel dose-dependent reductions in cell viability were observed in both pREP4 and FABP12+ PC3 cells, the effects on cell viability were considerably attenuated in FABP12+ PC3 cells (*p* < 0.0001) (Figure 4A).

**Figure 5:**
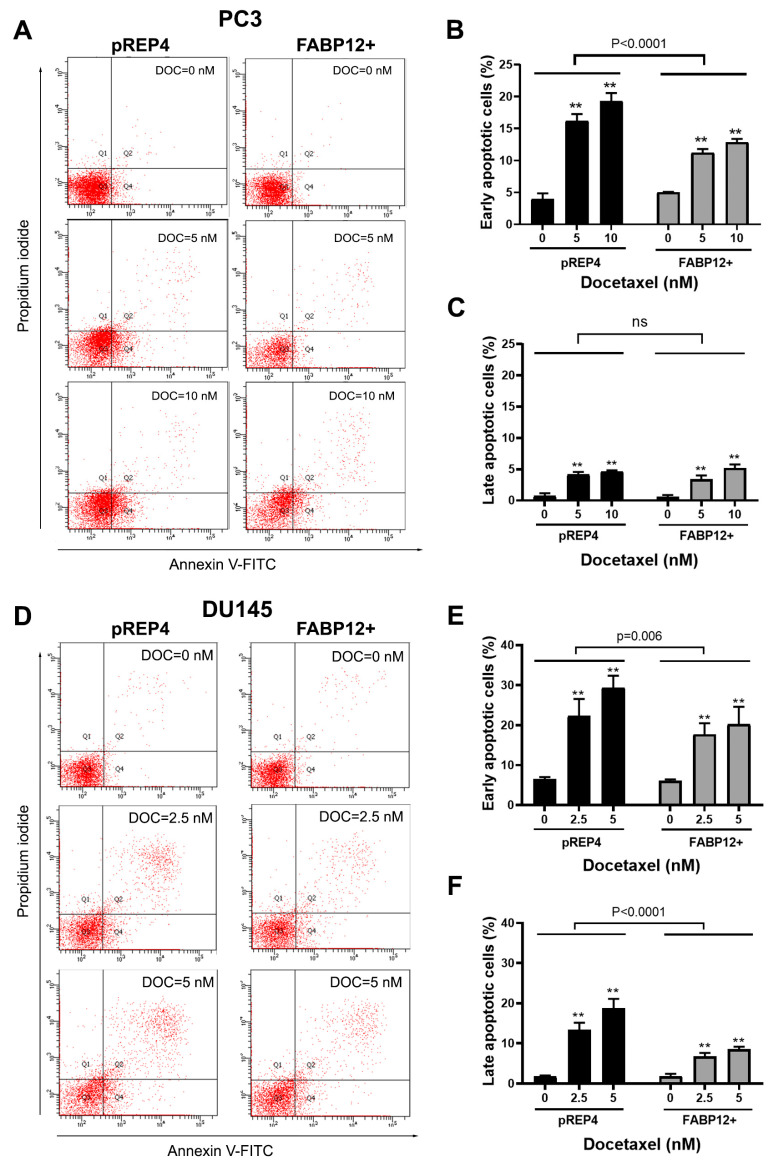
Effect of FABP12 expression on docetaxel-induced apoptosis. Untreated and docetaxel treated PC3 (**A**–**C**) and DU145 (**D**–**F**) stable transfectant cells (control pREP4 and FABP12+) were harvested and stained with Annexin V-FITC and PI according to the manufacturer’s protocol. Cells were analyzed using a flow cytometer to identify Annexin V-negative and PI-negative (viable or healthy cells; Quadrant 3 (Q3)), Annexin V-positive, PI-negative (early apoptosis; Q4), Annexin V-positive, PI-positive (late apoptosis; Q2) and Annexin V-negative, PI-positive (necrotic; Q1). (**A**) (PC3) and (**D**) (DU145) show representative dot plots for Annexin V-FITC and PI staining. Histograms show the quantification (in percentage) of early (**B**,**E**) and late (**C**,**F**) apoptotic cells based on Annexin V-PI staining. Experiments were repeated three times; ns denotes not significant; **, *p* < 0.001.

We then examined the effect of FABP12 on docetaxel-induced apoptosis in PC3 cells using Annexin V staining and flow cytometry. Cells were treated with 5 and 10 nM docetaxel for 72 h. A significant reduction in the percentage of early stage apoptotic cells (*p* < 0.0001) was observed in FABP12+ PC3 cells compared to pREP4 control cells (Figure 5A,B). In contrast to early stage apoptotic cells, we observed few cells in late stage apoptosis, with no difference in late stage apoptosis noted between pREP4 and FABP12+ PC3 cells treated with docetaxel (Figure 5A,C).

To further document the role of FABP12 in docetaxel-mediated apoptotic cell death, we examined the effect of ectopic FABP12 expression on a second prostate cancer cell line, hormone-insensitive DU145. Cell viability analysis using the MTS assay revealed robust resistance to cell killing by docetaxel in FABP12+ relative to pREP4 control DU145 cells (Figure 4B). Flow cytometry analysis generated similar results to those observed in PC3 cells, with FABP12 expression in DU145 cells providing significant protection from docetaxel-induced cell death by apoptosis (Figure 5D). Percentages of both early (*p* < 0.006) and late (*p* < 0.0001) apoptotic cells were significantly reduced in DU145 FABP12+ cells compared to pREP4 control cells (Figure 5E,F). To verify the effect of FABP12 on apoptosis, we carried out Western blotting of docetaxel-treated DU145 pREP4 and FABP12+ cells using an antibody to the apoptotic marker, cleaved PARP (c-PARP). Induction of cleaved PARP was considerably stronger in pREP4 cells treated with docetaxel compared to FABP12+ cells (Figure 4C). While muted compared to pREP4 cells, induction of c-PARP was also observed in FABP12+ cells treated with 5 nM docetaxel. Although Survivin was not detected in DU145 pREP4 cells (Figure 4C, lane 1), docetaxel treatment resulted in induction of Survivin levels in these cells. Similar to what we observed in PC3 cells, FABP12 expression in DU145 resulted in a dramatic upregulation in Survivin levels in untreated cells (Figure 4C, lane 4). No further increases in Survivin levels were observed upon docetaxel treatment in DU145 FABP12+ cells. These results are in agreement with FABP12 inhibiting docetaxel-induced apoptosis in PCa cells via the apoptosis inhibitor Survivin.

### 2.5. Survivin Is Associated with Fatty Acid-Binding Protein 12 (FABP12) and Patient Prognosis in Prostate Cancer

**Figure 6:**
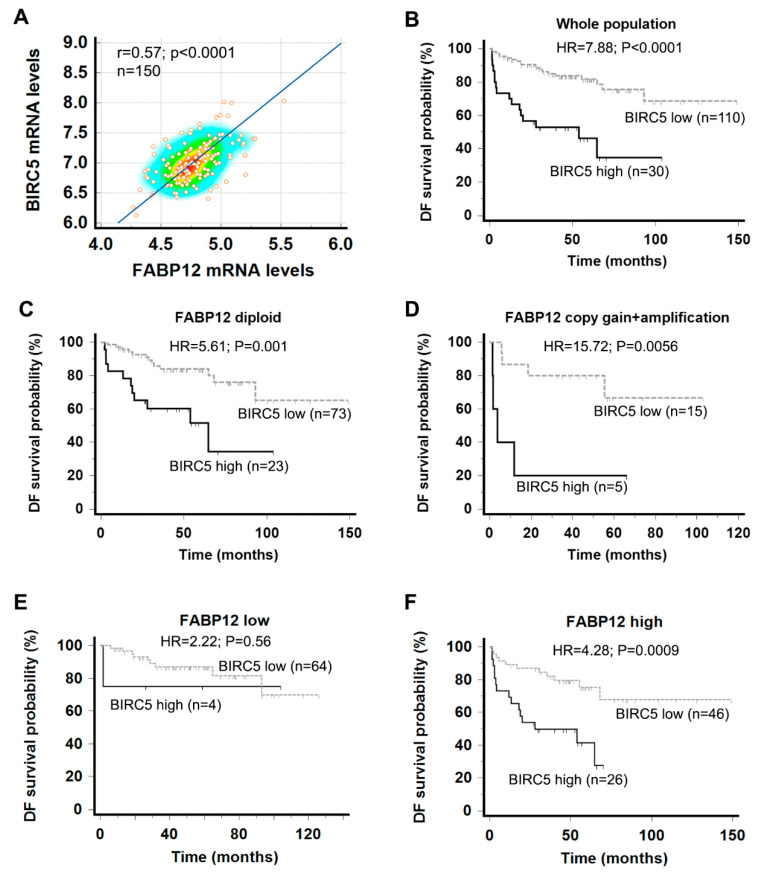
Prognostic significance of the Survivin gene *BIRC5* in PCa patients and its dependence on FABP12 status. (**A**) Correlation of *FABP12* mRNA levels with *BIRC5* mRNA levels in a PCa patient cohort [23]. (**B**) Kaplan-Meier patient survival curves in patient populations stratified based on high and low *BIRC5* mRNA levels. (**C**,**D**) Kaplan-Meier survival curves showing the differences in the prognostic significance of *BIRC5* based on *FABP12* gene copy numbers [diploid (**C**) vs. gene copy gain/amplification (**D**). (**E**,**F**) Kaplan-Meier survival curves showing the differences in the prognostic significance of *BIRC5* in sub-populations with low (**E**) or high (**F**) *FABP12* expression levels; r denotes correlation coefficient; n, sample size; HR, hazard ratio.

Our findings suggest that Survivin is a downstream effector of FABP12 in promoting resistance to docetaxel treatment. We next addressed a possible relationship between FABP12 and Survivin in PCa patient tissues, as well as their interactive implications in patient prognosis. For these analyses, we used a PCa patient cohort with documented *FABP12* RNA and DNA sequencing data [23]. We found a significant correlation between *FABP12* and *BIRC5* mRNA expression in this cohort (r = 0.57, *p* < 0.0001; Figure 6A). Similar to that previously reported for *FABP12* RNA in this PCa patient cohort [15], high levels of *BIRC5* mRNA were significantly associated with poor prognosis (HR = 7.88, *p* < 0.0001; Figure 6B). Remarkably, examination of *BIRC5*-low versus *BIRC5*-high subpopulations of PCa patients for gain or amplification of *FABP12* gene copy numbers (in a total of 20 patients) generated an HR of 15.72 (*p* = 0.006; Figure 6D). The HR was reduced to 5.61 in the more abundant diploid populations (96 patients) (*p* = 0.001; Figure 6C). When this cohort was stratified based on low and high *FABP12* expression levels, the association between BIRC5 and patient survival became insignificant in the subpopulation with low levels of FABP12 (HR = 2.22, *p* = 0.56; Figure 6E), but remained highly significant in the subpopulation with high levels of FABP12 (HR = 4.28, *p* = 0.0009; Figure 6F). These results provide further support for a functional relationship between FABP12 and Survivin as factors influencing PCa patient prognosis by promoting cell growth/survival and attenuating treatment efficacy.

## 3. Discussion

While there are many options for the treatment of primary PCa, systemic therapy is the preferred treatment approach when PCa progresses to castration resistance and metastatic disease. Docetaxel, a first-line chemotherapeutic drug used for the treatment of advanced PCa, has been shown to prolong survival in patients with metastatic PCa; however, resistance to docetaxel often develops over time [24,25]. Understanding the mechanism(s) underlying docetaxel resistance may lead to improved approaches for the treatment of PCa. In a previous report, we showed that FABP12, an EMT inducer and regulator of lipid-related bioenergetics, is preferentially amplified and enriched in metastatic PCa [15]. In the current study, we expand on these findings by demonstrating that FABP12 also plays an important role in docetaxel resistance in PCa cells. EMT and mesenchymal-to-epithelial transition (MET) are dynamic and reversible processes with critical physiological roles in embryogenesis and organ development [26]. As mesenchymal cell state is associated with the capacity of cells to migrate to distant organs and maintain stemness, cancer cells employ EMT to endow cells with migratory and invasive properties, to induce cancer stem cell properties, and to prevent apoptosis and senescence [26,27]. EMT is now thought to play a fundamental role in tumor progression and metastasis formation [28,29]. There is increasing evidence pointing to EMT as one of the key mechanisms underlying resistance to cancer treatment [8,9,30]. For example, EMT and metastasis-promoting factors ZEB1 and ZEB2 have been reported to act as mediators of docetaxel-resistance in PCa by promoting EMT [31]. 

In this manuscript, we report a functional link between FABP12, the EMT factor Slug and Survivin in driving docetaxel resistance in PCa cells. Survivin is a member of the inhibitor of apoptosis (IAP) family often overexpressed in cancers, including aggressive PCa [32]. Survivin has been shown to inhibit caspase-mediated apoptosis although it can also act independently of caspases [4]. Ectopic expression of FABP12 in PCa cell lines that normally express little if any FABP12 results in suppression of docetaxel-mediated inhibition of cell growth through increased cell viability and reduced apoptosis. Importantly, FABP12-dependent upregulation of Survivin in PCa cells is reversed by Slug depletion, suggesting a direct link between EMT and docetaxel resistance in FABP12-expressing PCa cells. The presence of a FABP12-Slug-Survivin pathway that promotes docetaxel resistance in PCa cells suggests possible avenues for the mitigation of docetaxel resistance in aggressive PCa. Both Survivin and Slug have already received considerable attention as possible therapeutic targets for aggressive cancers, including PCa [33,34]. As our FABP12 and Slug depletion experiments indicate that Survivin resides downstream of Slug, and Slug resides downstream of FABP12, we propose that addition of a FABP12 inhibitor to docetaxel treatment regimens may prevent or at least delay the development of docetaxel resistance in PCa cells.

**Figure 7:**
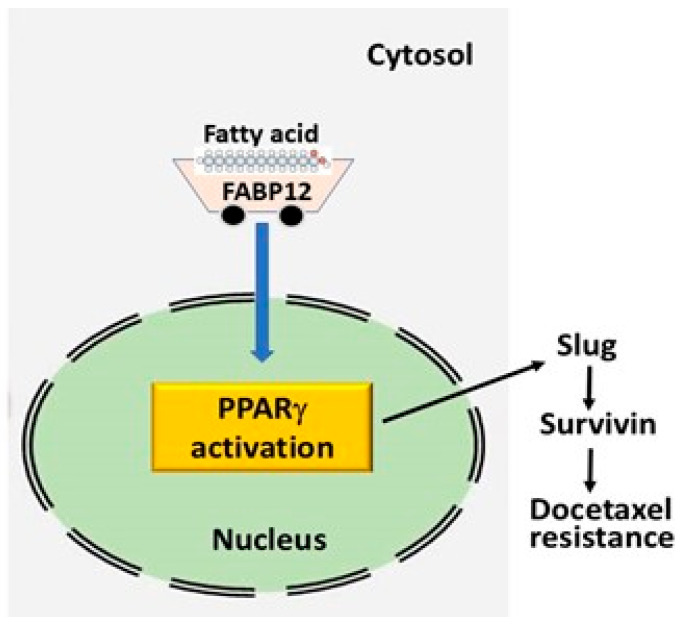
A schematic model to summarize the role of the FABP12-PPARγ-Slug-Survivin pathway in the regulation of docetaxel resistance in prostate cancer cells. FABP12, predicted to serve as an intracellular transporter of long chain polyunsaturated fatty acids (FAs), facilitates FA nuclear translocation, where it interacts and activates PPARγ. PPARγ then regulates the expression of Slug, likely through direct binding to its upstream promoter sequence. Slug, in turn, regulates expression of Survivin, leading to docetaxel resistance in PCa cells by suppressing docetaxel-induced apoptosis.

While there is no direct evidence to support interaction between PPARγ with the promoter region of *SNAI2* (the gene encoding Slug) thereby directly regulating its transcription, we have identified at least two PPARγ binding sites within 1000 bp of the *SNAI2* transcription initiation site using MatInspector [35]. We propose that FABP12 facilitates nuclear translocation of PUFA (e.g., arachidonic acid) and activates PPARγ. This, in turn, induces *SNAI2* transcription, leading to EMT and drug resistance (Figure 7).

FABPs are a family of ten proteins with distinct spatial and temporal distribution patterns. These small proteins serve as chaperones for the transport of different types of fatty acids and other lipids to different sites within the cell, including the nucleus [36]. While the fatty acid specificity of FABP12 remains to be investigated, in silico simulation of protein-ligand interaction suggests that omega-6 polyunsaturated arachidonic acid is a preferred ligand of FABP12. Arachidonic acid and some of its eicosanoid derivatives (e.g., prostaglandins) are well-known for their pro-inflammatory/pro-tumorigenic roles and have been implicated in PPARγ activation [37,38]. Thus, the effect of FABP12 on PCa lipid metabolism, metastasis, and treatment resistance may be at least partially mediated through binding of FABP12 ligands (or their metabolites) to PPARγ. FABP12 has a highly restricted tissue distribution, being primarily confined to cap phase spermatids and acrosome spermatids during spermatogenesis [15]. Targeting FABP12, as opposed to the more broadly-expressed Survivin, Slug or PPARγ, especially in patients with advanced or metastatic tumors, may therefore be of added benefit due to reduced side effects. 

There may be alternative FABP12-mediated mechanisms or pathways involved in cell response to docetaxel in PCa. For instance, aberrant lipid metabolism in cancers including PCa has been shown to contribute to tumor cell plasticity and drug resistance [7]. As well, we previously reported that FABP12 plays a critical role in regulating mitochondria β-oxidation and bioenergetics [15]. Thus, FABP12-mediated lipid metabolic pathways may also contribute to docetaxel resistance in PCa cells. Future work will involve further examination of the pathways affected by FABP12 overexpression in PCa. As FABP12 is a member of the FABP family consisting of 10 genes in mammals, it will be important to identify small molecule inhibitors that specifically target FABP12. In light of the paucity of established PCa cell lines expressing FABP12, this study was carried out using gain-of-function approaches. We will next focus on generating patient-derived organoids and xenografts from PCa patients to address the role of FABP12 and related pathways in PCa treatment resistance and progression using loss-of-function approaches.

In summary, we report that FABP12, previously shown to be associated with PCa progression and metastasis, confers resistance to the chemotherapeutic drug docetaxel in PCa cells. We provide insight into the mechanism of action of FABP12, showing that FABP12 expression induces the inhibitor of apoptosis, Survivin, which in turn protects FABP12-expressing PCa cells from undergoing apoptosis. We also show that Slug is implicated in the FABP12-Survivin axis, residing upstream of Survivin expression. Our research suggests that targeting FABP12 along with docetaxel treatment may help to overcome drug resistance in patients with advanced PCa.

## 4. Materials and Methods

### 4.1. Cell Culture and Docetaxel Treatment

The PC3 and DU145 cell lines were obtained from ATCC and frozen in liquid nitrogen within two passages of receipt. Cells were cultured in DMEM supplemented with 10% fetal bovine serum (FBS), 50 U/mL of penicillin and 50 µg/mL of streptomycin, and in a humidified environment containing 5% CO_2_ at 37 °C. Docetaxel was purchased from Sigma-Aldrich (St. Louis, MO, USA) (CAS number: 114977-28-5) and dissolved in dimethylsulfoxide (DMSO) and stored in small aliquots at −20 °C. Cells were treated with docetaxel at the indicated concentrations in antibiotics-free DMEM containing 10% FBS.

### 4.2. Stable Transfection of Fatty Acid-Binding Protein 12 (FABP12) in Prostate Cancer Cells

For FABP12 ectopic expression, stable cell lines were generated by transfecting PC3 or DU145 cells with pREP4 or pREP4-FABP12 using the polyethylenimine (PEI) reagent, followed by selection in hygromycin (100 µg/mL). Individual colonies of transfected cells were picked using cloning rings and expanded. The PC3 clonal populations are described in Liu et al. [15]. We chose the PC3 and DU145 cell lines for our analyses because they express PPARγ, the key player meditating FABP12 function in PCa [15]. Importantly, the parental cell lines (PC3 and DU145) and pREP4-transfected PC3 and DU145 (control) cells share similar gene expression profiles (e.g., FABP12, Slug, Survivin, etc.) and morphological characteristics.

### 4.3. siRNA Transfection

For knockdown experiments, stable PC3-pREP4 (control) and PC3-pREP4-FABP12 (FABP12+) cells were transfected with 10 nM scrambled (siControl) or specific siRNAs targeting *SNAI2* (encoding Slug), *PPARγ* or *FABP12* (Life Technologies Inc., Burlington, ON, Canada; sequences listed in Supplementary Table S1) using the RNAiMAX transfection reagent (Thermo Fisher Scientific, Waltham, MA, USA) following the manufacturer’s protocol. 

### 4.4. Cell Growth and Viability Assays

For cell growth, stably transfected PC3 cells were seeded in 96-well plates (1500 cells/well) and cultured for 48 h. Culture medium was then replaced with antibiotic-free DMEM containing 10% FBS and docetaxel in gradient concentrations. At the specified time points (24, 48, or 72 h), the medium was removed and cells washed twice with PBS and stained with crystal violet. Cell density was analyzed with a FLUOstar Omega microplate reader (BMG Labtech, Ortenberg, Germany). To measure cell viability, 20 µL of MTS solution (Promega, Madison, WI, USA) were added to each well of the 96-well plates containing 100 µL of culture medium with or without docetaxel and incubated for 4 h. Cell viability was determined by measuring absorbance at 490 nm using the FLUOstar Omega (BMG Labtech, Ortenberg, Germany) microplate reader.

### 4.5. Western Blotting

Cell lysates were prepared as previously described [39]. Western blot analysis was carried out using 40 μg whole cell lysates per lane. Proteins were separated by SDS-PAGE and electroblotted onto nitrocellulose membranes. Blots were immunostained with primary antibodies diluted with 5% BSA in 1X TBS (Supplementary Table S2). Primary antibodies were detected with horseradish peroxidase-conjugated secondary antibodies and visualized using the ECL detection system (GE Healthcare Life Sciences, Chicago, IL, USA) or SuperSignal™ West Pico PLUS Chemiluminescent Substrate (Thermo Fisher Scientific, Waltham, MA, USA).

### 4.6. RT-PCR

Total RNA was isolated using the TRIzol reagent (Invitrogen, Life Technologies, Carlsbad, CA, USA). First strand cDNAs were synthesized using SuperScript reverse transcriptase II (Invitrogen) according to the protocol provided by the manufacturer. Oligonucleotide primers used for PCR amplifications are listed in Supplementary Table S3. PCR reactions and agarose gel electrophoresis were carried out as previously described [39]. 

### 4.7. Annexin V-PI Staining and Flow Cytometry

Docetaxel-induced apoptosis in PC3 and DU145 stable transfectants was analyzed using the FITC Annexin V Apoptosis Detection Kit I (BD Biosciences, Franklin Lakes, NJ, USA). Cells were treated with docetaxel (at the indicated concentrations) for 72 h or left untreated. Untreated and treated cells were harvested, washed with PBS, and stained with Annexin V-FITC and propidium iodide (PI) in 1X binding buffer for 15 min. Unstained cells were used for autofluorescence. Cells stained with either Annexin V only or PI only were used to compensate for channel spillover. Cell suspensions were diluted with 1X binding buffer and analyzed using a flow cytometer (FACS Canto II, BD Biosciences, Franklin Lakes, NJ, USA) to identify the following cell populations: FITC-negative and PI-negative (viable or healthy cells; quadrant 3), FITC-positive and PI-negative (early apoptosis; quadrant 4), FITC-positive and PI-positive (late apoptosis; quadrant 2), or FITC- negative and PI- positive (necrosis; quadrant 1). Gating for quadrant 3 (lower left) was adjusted to retain most of the Annexin V-negative (FITC-negative) and PI-negative cells from untreated samples. Cells with FITC fluorescence intensity higher than the background fluorescence of untreated cells were considered as FITC-positive and cells with PI fluorescence higher than the fluorescence of untreated cells were considered as PI-positive. Experiments were repeated three times and the observed values were averaged.

### 4.8. Statistical Analysis

Two-way ANOVA was used to test the statistical significance of effects (variation) derived from experimental factors (e.g., FABP12 status, drug treatment) and their interactive effects. The multiple comparison module was used to show significance of difference between control and drug-treated cells within a single factor (e.g., FABP12 overexpression or Slug knockdown). Linear correlation between the expression levels of two genes from a PCa patient cohort [23] was analyzed using the Pearson correlation test. Disease-free survival curves were generated by the Kaplan-Meier estimation and the survival probabilities between patient groups with high or low *BIRC5* (encoding Survivin) levels were compared by the log rank test using MedCalc Statistical Software version 22.021 (MedCalc Software Ltd., Ostend, Belgium). A *p* value < 0.05 was considered statistically significant.

## Data Availability

The data presented in this study are available in the article.

## Supplementary Materials

The following are available online at https://www.mdpi.com/article/10.3390/ijms25179669/s1.
