# Supplementary material for: Docetaxel-Induced Cell Death Is Regulated by a Fatty Acid-Binding Protein 12-Slug-Survivin Pathway in Prostate Cancer Cells
Source: Int J Mol Sci. 2024 Sep 6;25(17):9669. doi: 10.3390/ijms25179669 (PMC11395974; doi:10.3390/ijms25179669)
Supplement: Supplementary file 1 [file ijms-25-09669-s001.zip › ijms-3138544-supplementary.pdf]

Table S1. Sequences of siRNAs used for RNA depletion experiments

Gene symbol	siRNA-1	siRNA-2
FABP12	5’-CAAGGAACAUGGAAGUCCAUUUCUU-3’	5’-AAGAAAUGGACUUCCAUGUUCCUUG-3’
SNAI2	5’-AUCAGAAUGGGUCUGCAGAUGAGCC-3’	5’-CAGCUGCACUGCGAUGCCCAGUCUA-3’
PPARγ	5’-CAUACUUGUAAUCUGCAACCACUGG-3’	5’-AGGGAGUUUCUAAAGAGCCUGCGAA-3’

Table S2. List of antibodies used for western blotting

Antibody	Company	Purpose	Dilution	Secondary antibody
FABP12	In house-generated (DSHB)	Western Blotting	1:350	Rabbit (1:20,000)
Slug	Cell Signaling Technology	Western Blotting	1:1000	Rabbit (1:20,000)
Survivin	Santa Cruz Biotechnology, INC.	Western Blotting	1:100	Mouse (1:20,000)
Cleaved PARP	Cell Signaling Technology	Western Blotting	1:1000	Rabbit (1:25,000)
GAPDH	Invitrogen	Western Blotting	1:5000	Mouse (1:20,000)
beta-Actin	Sigma-Aldrich	Western Blotting	1:100,000	Mouse (1:25,000)

Table S3. Sequences of PCR primer pairs used for RT-PCR

Gene symbol	Forward primer	Reverse primer
FABP12	5'-TCTGAGACAGAATGATTGACC-3'	5’-GTCAGTAGTTTGGATGACAGC-3’
BIRC5	5'-GCCATTAACCGCCAGATTTG-3'	5'-TTCTTGGCTCTTTCTCTGTCC-3'
ACTB (actin)	5'-CTGGCACCACACCTTCTAC-3'	5'-CATACTCCTGCTTGCTGATC-3'
